# Editorial: The operationalization of cognitive systems in the comprehension of visual structures

**DOI:** 10.3389/fnins.2024.1501636

**Published:** 2024-10-07

**Authors:** Michael Winter, Thomas Probst, Miles Tallon, Johannes Schobel, Rüdiger Pryss

**Affiliations:** ^1^Institute of Clinical Epidemiology and Biometry, University of Würzburg, Würzburg, Germany; ^2^Institute of Medical Data Science, University Hospital of Würzburg, Würzburg, Germany; ^3^Division of Psychotherapy, Department of Psychology, Paris Lodron University Salzburg, Salzburg, Austria; ^4^HSD Hochschule Döpfer, University of Applied Sciences, Köln, Germany; ^5^DigiHealth Institute, Neu-Ulm University of Applied Sciences, Neu-Ulm, Germany

**Keywords:** cognitive systems, visual comprehension, working memory, neural mechanisms, cognitive load, event-related potential, object recognition

## 1 Introduction and aims of the Research Topic

This Research Topic brings together a collection of studies that discuss the complex relationships between cognitive processes and visual perception. Visual comprehension is a multifaceted cognitive task that necessitates the integration of attention, memory, and sensory information to form coherent representations of the visual world (Wyer et al., 2008). These processes involve not only low-level perceptual mechanisms but also higher-order cognitive functions, such as decision-making and problem-solving, making it a vital area of study across disciplines (Jarecki et al., 2020). Understanding how the brain orchestrates these functions is essential for advancing research in fields such as neuroscience, psychology, artificial intelligence, and clinical applications (Bryck and Fisher, 2012). Moreover, research in visual comprehension has implications for the development of AI systems designed to mimic human-like perception and for creating clinical interventions aimed at enhancing cognitive function in individuals with impairments (Yildirim et al., 2024).

The aim of this Research Topic was to investigate how cognitive systems (e.g., attention and perception) operationalize the comprehension of visual structures, focusing on neural mechanisms, cognitive load, and memory processes. The contributing studies provide insights into the interaction between visual working memory, attention, and postural control, as well as the neural correlates of object recognition. These findings not only advance theoretical models of visual cognition but also offer potential applications in technology and clinical practice.

## 2 Key contributions of the Research Topic

The four articles within this Research Topic offer insights into the operationalization of cognitive systems in visual comprehension, each addressing unique aspects of visual memory, attention, and neural processing.

### 2.1 Memory impairment and attention deficits in frontal lobe epilepsy

The first article examines the cognitive impact of frontal lobe epilepsy (FLE) on memory and attention using a novel eye-tracking paradigm (Zhang et al.). The results show that FLE patients experience deficits in short-term memory, particularly during the retrieval phase. Eye-tracking data reveals prolonged fixation times and reduced visual attention efficiency, linking these impairments to difficulties in sustaining attention. The study highlights eye-tracking's potential in distinguishing attention deficits from memory dysfunctions in FLE patients.

### 2.2 Exploring retro-cue effects on visual working memory

The second contribution examines the effects of retro-cues on visual working memory (VWM), focusing on the benefits (RCB) and costs (RCC) of double-cue paradigms (Guo et al.). The findings show that while double cues improve memory performance over neutral cues, they are less effective than single cues. EEG data reveal that uncued items remain passive until reactivated by a second cue, emphasizing the dynamic nature of memory storage. This research provides insights into how internal attention shifts affect VWM, advancing understanding of cognitive resource allocation during complex visual tasks.

### 2.3 Cognitive load and postural control in visual working memory

The third article explores the relationship between visual working memory and upright postural control using an event-related potential (ERP) approach in the n-back paradigm (Wei et al.). The findings show that cognitive load from visual memory tasks affects postural control, leading to increased sway in more demanding tasks. ERP data indicate that while upright posture enhances early selective attention, it interferes with later memory encoding. These results highlight the competition for neural resources between physical balance and cognitive tasks, offering insights into dual-task processing in daily life.

### 2.4 Neural indicators of cognitive load during visual search tasks

The fourth contribution examines how cognitive load affects neural activity during visual search tasks, using event-related potentials (ERPs) as indicators (Shan et al.). The study shows that higher cognitive load reduces ERP components like P300 amplitude, indicating greater difficulty in attention allocation and memory processing. These findings provide insights into the neural mechanisms of attention and cognitive load, suggesting that the brain's capacity for visual search tasks decreases as cognitive demands rise. This research enhances understanding of how neural efficiency is impacted by complex cognitive tasks, with potential applications in clinical and technological fields.

## 3 Broader context and thematic connections

The articles in this Research Topic advance our understanding of how cognitive systems manage visual comprehension, focusing on key themes like cognitive load, attention, memory, and neural efficiency. A central theme is the competition for cognitive resources in tasks combining postural control and visual memory, highlighting how the brain allocates resources under demand. These studies underscore the role of selective attention, linking it to neural markers like P300, and show how factors like posture influence performance.

Neural adaptability is also emphasized, particularly in studies on cognitive load and object recognition, offering insights into how the brain maintains perception in dynamic environments. Eye-tracking data further sheds light on how conditions like frontal lobe epilepsy affect cognitive function. Importantly, these studies suggest that cognitive performance is not only task-dependent but also highly influenced by the interaction between environmental demands and the individual's internal state. These findings have implications for developing AI systems that mimic human visual processing and inform clinical interventions for cognitive impairments in conditions such as dementia, brain injury, and epilepsy.

## 4 Future research directions

The findings in this Research Topic pave the way for future exploration, particularly in understanding how cognitive systems manage visual comprehension under various conditions. Future research could focus on cognitive resource allocation in complex, real-world environments with multiple sensory inputs and physical demands, shedding light on performance variability in multitasking. Longitudinal studies could also reveal whether sustained cognitive load enhances neural efficiency or leads to fatigue. Research on dual-task interference, especially with tasks involving both cognitive and physical components, would benefit from advanced neuroimaging techniques to better understand how the brain manages resource competition.

These insights have practical applications in AI, where attentional control and memory prioritization could improve computer vision systems. Clinically, they could inform interventions for cognitive impairments, using techniques like neurofeedback and cognitive training to enhance visual and memory functions. Additionally, understanding how cognitive systems adapt under high cognitive load can offer strategies to mitigate cognitive fatigue in high-stress occupations or everyday multitasking scenarios. Exploring the interaction between emotions and cognitive load could further enrich our understanding of how cognitive systems integrate emotional and perceptual information under demanding conditions.

## 5 Summary

This Research Topic examines the interactions between cognitive processes like attention, memory, and neural adaptation in visual comprehension. The articles explore key themes such as the competition for cognitive resources, the role of attention in working memory, and neural mechanisms behind cognitive load during multitasking and object recognition. These studies enhance our understanding of how cognitive systems manage visual information, with implications for AI development and clinical rehabilitation. Future research suggests exploring these findings in more complex, real-world environments, and investigating the role of emotions in cognitive load.

## References

[B1] Bryck R. L. Fisher P. A. (2012). Training the brain: practical applications of neural plasticity from the intersection of cognitive neuroscience, developmental psychology, and prevention science. Am. Psychol. 67:87. 10.1037/a0024657 21787037 PMC3335430

[B2] Jarecki J. B. Tan J. H. Jenny M. A. (2020). A framework for building cognitive process models. Psychon. Bull. Rev. 27, 1218–1229. 10.3758/s13423-020-01747-2 32632887 PMC7704479

[B3] Wyer Jr R. S. Hung I. W. Jiang Y. (2008). Visual and verbal processing strategies in comprehension and judgment. J. Cons. Psychol. 18, 244–257. 10.1016/j.jcps.2008.09.002

[B4] Yildirim N. Richardson H. Wetscherek M. T. Bajwa J. Jacob J. Pinnock M. A.. (2024). “Multimodal healthcare AI: identifying and designing clinically relevant vision-language applications for radiology,” in Proceedings of the Conference on Human Factors in Computing Systems (CHI), 1–22. 10.1145/3613904.3642013

